# An implantable neurophysiology platform: Broadening research capabilities in free-living and non-traditional animals

**DOI:** 10.3389/fncir.2022.940989

**Published:** 2022-09-23

**Authors:** Matt Gaidica, Ben Dantzer

**Affiliations:** ^1^Department of Psychology, University of Michigan, Ann Arbor, MI, United States; ^2^Department of Ecology and Evolutionary Biology, University of Michigan, Ann Arbor, MI, United States

**Keywords:** physiology, sleep, accelerometer, closed-loop, wireless, implantable

## Abstract

Animal-borne sensors that can record and transmit data (“biologgers”) are becoming smaller and more capable at a rapid pace. Biologgers have provided enormous insight into the covert lives of many free-ranging animals by characterizing behavioral motifs, estimating energy expenditure, and tracking movement over vast distances, thereby serving both scientific and conservational endpoints. However, given that biologgers are usually attached externally, access to the brain and neurophysiological data has been largely unexplored outside of the laboratory, limiting our understanding of how the brain adapts to, interacts with, or addresses challenges of the natural world. For example, there are only a handful of studies in free-living animals examining the role of sleep, resulting in a wake-centric view of behavior despite the fact that sleep often encompasses a large portion of an animal’s day and plays a vital role in maintaining homeostasis. The growing need to understand sleep from a mechanistic viewpoint and probe its function led us to design an implantable neurophysiology platform that can record brain activity and inertial data, while utilizing a wireless link to enable a suite of forward-looking capabilities. Here, we describe our design approach and demonstrate our device’s capability in a standard laboratory rat as well as a captive fox squirrel. We also discuss the methodological and ethical implications of deploying this new class of device “into the wild” to fill outstanding knowledge gaps.

## Introduction

Over the past 100 years, behavioral and physiological research has undergone a striking technological progression. Increasingly, microscopes, binoculars, and restrictive manipulandum are being replaced by small electronics that promote freedom of movement while describing internal and external animal states with unprecedented accuracy (Wilson et al., 2008; Wilmers et al., 2015; Williams et al., 2020). Major advances were pioneered in aquatic birds, demonstrating the utility of hermetically sealed “biologgers” to characterize subsurface porpoising and diving behavior (Butler and Woakes, 1979; Yoda et al., 1999). Breakthroughs in component miniaturization, computational capacity, and low-power optimization have all contributed to the modern form factor and capability of biologgers and similar animal-borne telemetry systems (Wilmers et al., 2015; Harcourt et al., 2019; Whitford and Klimley, 2019). For example, spatially resolute wireless tracking systems have been used to investigate social complexity in bats (Ripperger et al., 2016, 2019), understand nesting in starlings (Kirkpatrick et al., 2021), and pave the way for characterizing complex pair-bonding relationships. Small GPS loggers have utilized novel satellite networks to research migratory patterns of birds (Jetz et al., 2022) and were key to identifying the single longest migration on record in a gray whale (Mate et al., 2015). Increasingly, technology is enabling a better understanding of how animals interact with their natural environment to serve fundamental scientific and conservational endpoints (Chmura et al., 2018; Jetz et al., 2022).

Despite major innovations, a species-wide gap remains in using biologgers to understand neural physiology outside of the laboratory (Rattenborg et al., 2017). Behavior restricted by neural recording techniques that require a tether or enclosed recording chamber (Aulehner et al., 2022), and the removal of natural cues regulating sleep-wave cycles (called, *Zeitgebers*) such as light, temperature, social interaction, and resource availability may drastically affect, or even nullify, physiological interpretations of such data. In particular, understanding the natural transitions between rest and activity (or vice versa), and the complex architecture of neural states within sleep itself, represents a major barrier in describing the function and evolutionary process of sleep (Roth et al., 2010; Aulsebrook et al., 2016; Eban-Rothschild et al., 2017; Anafi et al., 2019). For example, discovering that birds sleep mid-flight (Rattenborg et al., 2016) or that some animals sleep one hemisphere at a time (Mascetti, 2016) is contrary to our conventional understanding of how sleep and movement are related. Sleep remains an enigmatic quiescent state that plays a number of vital roles, both for individuals and groups (Mistlberger and Skene, 2004). Although accelerometers have been widely deployed and repurposed to meet challenges in quantifying sleep, they are fraught with validation issues in animals that are not easily observed (Gaidica et al., 2021). For example, Loftus et al. (2022) used collar-mounted accelerometers to determine sleep but ultimately failed to find evidence for a change in sleep intensity after sleep deprivation in wild non-human primates. This finding is inconsistent with decades of research suggesting that electrophysiological “deep” sleep has a rebounding effect to support homeostasis (Rechtschaffen, 1998), calling to question the utility of collar (or wrist) mounted inertial sensors.

Progress toward recording or transmitting neural activity in free-ranging animals has been made using small, externally fitted biologgers (Vyssotski et al., 2006; Rattenborg et al., 2008; Hoffmann et al., 2019; Massot et al., 2019). However, these devices are limited to species and environments where attaching the device directly to the head does not threaten naturalistic behaviors, such as entering and exiting burrows or nests. Therefore, until similar devices can be embedded within the organism and subsequently removed without consequence, the repertoire of species for which neural data (which provides crucial information about sleep) can be collected will be inherently limited (Forin-Wiart et al., 2019). To that end, implantable telemetry systems that require nearby antennas offer a glimpse into how such a feat can be accomplished (Chang et al., 2011), but at present, fully “autonomous” solutions are lacking.

**FIGURE 1:**
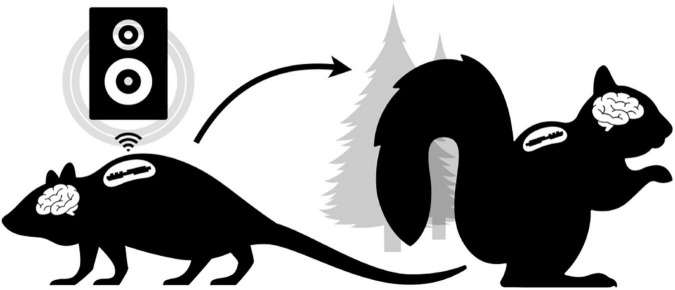
Conceptual Overview. The laboratory setting (left) does not have tools to enable freely behaving neurophysiology in novel experimental paradigms (e.g., low-latency wireless cuing of an external speaker to modulate neural rhythms in real-time). Ideally, the same “biologger” platform can translate to ethical free-ranging experiments (right) utilizing alternative device modes that support autonomous deployment.

In this study, we demonstrate progress toward a bridge between laboratory and field science that can be applied across species. Specifically, we developed an implantable biologging platform capable of broadening the understanding of neurophysiology and behavior in freely moving animals (Figure 1). Our biologger is based around a low-cost, highly capable Bluetooth Low Energy (BLE; see Table 1) microprocessor that schedules onboard recording of biopotential data. We first explored the general constraints and possibilities of embedded BLE technology by using our biologger to perform closed-loop audio stimulation in a freely behaving rat, a technique that could be used to enhance slow-wave (SW) neural rhythms that occur in NREM sleep (Bellesi et al., 2014) or tied together with other behavioral paradigms. Next, we demonstrated the utility of an implantable device by recording sleep in a captive squirrel from the comfort of their nest box. We showcase several important advancements in biologging, including sterile best practices, the ability for “wild” animals to survive the biologger implant and explant processes, and the increased capability of a platform that can be reconfigured in real-time and repurposed for multiple deployments. Although we focused on neurophysiology, the onboard accelerometer make more conventional insights into animal movement and behavioral motifs possible (Chimienti et al., 2016; Hammond et al., 2016). Finally, our biologger’s native wireless capability enables emerging use cases (e.g., proximity logging and spatial trilateration) to address further gaps in neurophysiological and behavioral research.

**TABLE 1:** Glossary.

Term	Definition
Bluetooth low energy (BLE)	A wireless network technology operating in the 2.4 GHz band standardized by the Bluetooth Special Interest Group.
BLE: central device	A device that connects to many peripheral devices often in charge of reading, writing, or receiving notifications/indications of BLE characteristics.
BLE: peripheral device	A device that advertises a service with many individual characteristics (i.e., pieces of data).
BLE: advertising	Advertisements contain limited information and are required to initiate a BLE connection.
BLE: service	Services can have many characteristics (e.g., a car dashboard service may contain characteristics for speed and fuel).
BLE: characteristic	Characteristics describe and house a single piece of variable-length data. These can be read from, written to, notify (without read-receipt), or indicate (with read-receipt).
BLE: latency	Latency is the time it takes for data to wirelessly transfer. This can be modified by the central-peripheral connection settings and is inherently limited by random delays (<10 ms) in the BLE protocol to avoid data collisions.
Printed circuit board (PCB)	A multilayer, precisely machined epoxy resin laminate board that is computer-designed and connects soldered components.
Biopotential	Any biological signal detectable by means of recording a difference in voltage between two leads.
Electroencephalography (EEG)	Biopotentials detected from outside the brain, typically representing cortical neural activity.
Light emitting diode (LED)	Small lights that can be soldered to a PCB.
Slow-wave activity (SWA)	A neural biopotential typically observed during non-rapid eye movement sleep characterized by slow (0.5–4 Hz), large amplitude oscillations over the prefrontal cortex.

## Materials and methods

In this section, we outline the biologger hardware and software architecture (see section “Hardware and software architecture”), biological interface (section “Biological interface”), surgical procedures (section “Surgical procedures”), and metrics and statistics (section “Metrics and statistics”) presented in our results.

### Hardware and software architecture

#### Biologger hardware

**FIGURE 2:**
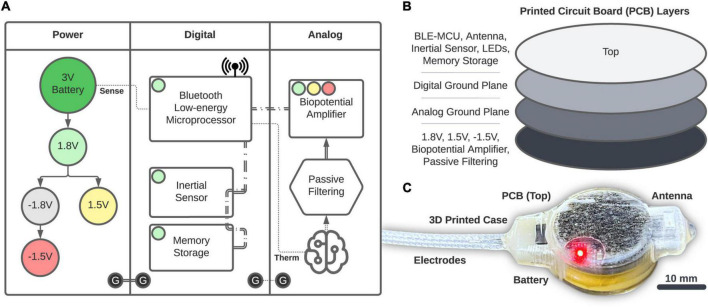
Biologger hardware. **(A)** High-level overview of the power, digital, and analog systems. **(B)** Component placement relative to the four-layer PCB. **(C)** The 3D-printed case with the PCB and electrodes encapsulated in silicone.

Power is provided by a 20 mm, 3 V coin-cell battery (2032, Energizer) that is fed into a series of voltage regulators (Figure 2A). The 1.8 V regulation (TPS62243, Texas Instruments) provides the main power rail for all components and is then inverted (MAX1720, Analog Devices) and regulated to −1.5 V (TPS72301, Texas Instruments) and 1.5 V (TPS7A2015, Texas Instruments) to provide a bipolar source. Digital components consist of the Bluetooth Low-energy (BLE, Version 5.2) Microprocessor (MCU; CC2652R, Texas Instruments) that performs logic, computation, and wireless communication through a single chip RF Balun (2450BM14G0011, Johanson Technology) and 2.4 GHz chip antenna (2450AT42B100, Johanson Technology). The BLE-MCU passively senses battery voltage and an optional external thermistor (GA100K6A1IA, TE Connectivity; single-dotted lines), as well as communicates across two, 3-wire SPI buses (double-dashed lines). The first SPI bus accesses a 6-axis accelerometer and gyroscope inertial sensor (LSM6DSOXTR, STMicroelectronics) and 2 Gb of NAND memory storage (MT29F2G01, Micron). The second SPI bus accesses the Analog section consisting of a 4-channel, 24-bit analog front-end biopotential amplifier (ADS1294, Texas Instruments) which collects bipolar biopotentials through an onboard passive filter network (0.25 Hz high-pass and antialiasing) and is available through a 1.27 mm-spaced pad array outfitted with a gold pin solder connector (851-43-008-10-001000, Mill-Max). Digital grounds are planar and tied to the analog circuits through a ground star. The printed circuit board (PCB; manufactured by PCBONLINE) has four layers (1 oz copper, 0.062” total thickness; Figure 2B) and was designed using KiCAD. We attempted to separate digital and analog components between the middle layers, and ground planes were structured according to manufacturer recommendations. All components (Figure 2C) were encased in 3D-printed biocompatible acrylic (Stratasys J750 with M3 crystal resin, designed with Autodesk Fusion 360). Electrode fabrication and silicone encapsulation are covered in detail below. The gross weight of the biologger was approximately 8.5 g (34% battery, 24% PCB, 22% 3D-printed case, and 20% epoxy and silicone).

A robust battery connection was required for this application. Metallic batteries can be heat damaged by soldering directly to their surface, and thus, we welded our own tabs to each side using a miniature spot welder (ıSW-001, Montex). Once welded, the cathode (positive) tab was directly soldered (X-TRONIC 5000 rework station, AIM 0.015” solder) to the biologger using a specially designed PCB pad, and the anode (negative) tab was connected to an underlying PCB pad using a short length of wire (26 gauge, Adafruit Industries). All soldering was performed with a filtered fume extractor (FES150, KNOKOO) and washed using flux remover (4140A, MG Chemicals).

#### Biologger software

Biologger software was developed in the C programming language using the Texas Instruments SDK (Version 5.2) within Code Composer Studio. Programming was made possible using a Texas Instruments development board (LP-CC2652RB) and a custom circuit board interface to supply power and communication to an 8-pin programming port on the biologger. The native Texas Instruments power policy was used to achieve low-power idle modes which automatically engage between application tasks.

Onboard biopotential filtering was achieved using the ARM-based CMSIS digital signal processing library and biquad cascade IIR filters. Bandpass filters coefficients were designed using ASN Filter Designer (Advanced Solutions Nederland B.V.).

The biologger runs some utilities regardless of the experiment, including a 1-s timer that maintains time and records biologger voltage, thermistor temperature, and the current time every 60 s for *post hoc* retrieval. Time is also logged during specific user actions, such as when settings or operating modes are changed. If battery voltage is less than 2.4 V the biologger settings are reset to defaults and writing to memory is halted to avoid undefined behavior that occurs outside of normal operating specifications. If the onboard memory is full, further writes will be rejected. Given these constraints, the bio-logger will eventually drain itself and cease to operate in a graceful manner. Specific functions initiated by the user include a recording scheduler that subsamples accelerometer (1 and 10 Hz options) and biopotential (125 Hz) data at regular intervals (e.g., record for 5 min every 60 min) and a closed-loop system which is described in detail below.

All data is stored in non-volatile (i.e., to persist without power) NAND memory in a serial fashion as self-contained, 32-bit packets. The first 8 bits encode a data type, and the remaining 24 bits encode data. This method makes data retrieval standard and robust, removing the overhead of implementing a filesystem that can be easily corrupted. Furthermore, although a filesystem has compression capabilities, should the biologger ever be damaged or undergo a catastrophic failure it is likely that partial data can be recovered if the memory module is intact.

#### Biologger configuration utility

**FIGURE 3:**
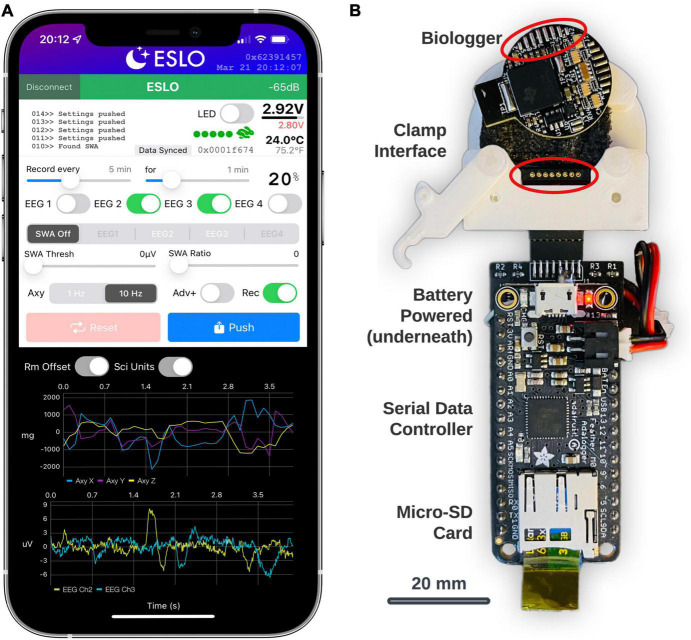
Biologger iOS app configuration utility and data dump module. **(A)** The app is shown in a connected state with a biologger. These settings represent a recording schedule mode where two channels of biopotentials (EEG2 and EEG3) will record with a 20% duty cycle and accelerometer (Axy) data will record at 10 Hz. **(B)** The data dump module is shown below the biologger interface. The bottom of the biologger is displayed here to appreciate how the programming port pins interface with the pogo connector (circled in red) in the custom clamp module. Data from the biologger memory is transferred serially through the main data controller board on the micro-SD card.

In general, custom BLE service characteristics enable the transfer of data, define the biologger state, and scheduled routines. The primary interface to control settings and view streaming data were built using Xcode (Version 13) using the Swift programming language. The app (called, “ESLO”) was deployed to an iPhone 12 Pro Max (Figure 3A). Biologgers were detected and connected based on a static address pattern. The app syncs date-time information to the biologger’s internal clock and indicates vital information (Figure 3A, upper-right): signal strength, battery voltage, thermistor temperature, motion activity, and current memory storage address. A built-in terminal area (Figure 3A, upper-left) allows for debugging messages to be printed and exported following a connection. Recording schedules, biopotential channels (labeled “EEG”), closed-loop controls (labeled “SWA”), and accelerometer modes and advertising modes (“Adv+”) are modified by the middle control set. Settings are synced from the biologger upon connecting and then manually “pushed” to the biologger after being modified. A reset button sets the biologger memory address to zero and also initiates “shelf mode” (see operating modes in Table 2). The dark panel (bottom) streams real-time data from the biologger: shown are 3-axes of accelerometer data and 2 EEG/biopotential channels. Centering data around zero (“Rm Offset”) and use of scientific units (“Sci Units”) can be toggled. A biologger can only be connected to one central device to avoid conflicts. Once the iOS app disconnects, the biologger will behave according to user settings. Subsequent connections to the biologger can be made based on the advertising interval, which defaults to 500 ms, but can be extended to a random interval of 30–60 s (“Adv+”) to decrease advertising power and provide a non-overlapping beacon if multiple biologgers are deployed in the same area.

**TABLE 2:** Operating modes and power estimates.

Mode	Description	Current	Power	Biologger lifetime
Shelf	BLE radio is off until the biologger detects significant motion (“shake to wake”)	44 μA	79.2 μW	6.8 years
Beacon	Biologger advertises its BLE service and becomes connectable every 30–60 s	0.5 mA	0.9 mW	40 days
Connected	Biologger is actively connected to a central device (e.g., iOS app)	2.3 mA	4.14 mW	4.3 days
Biopotentials	All 4 biopotential channels are on with 1 Hz accelerometer	2.8 mA	5.04 mW	3.5 days
Recording Ex. 1	2 biopotentials, 1 Hz accelerometer record 1 min every 5 min (20% duty cycle)	0.48 mA	0.86 mW	20 days
Recording Ex. 2	10 Hz accelerometer is always recording	130 μA	234 μW	76 days

All estimates are based on a 2032 battery (3 V, 240 mAh) operating at 85°F.

#### Biologger data retrieval

The biologger has an 8-pin programming and communication port where a “data dump” can occur using a standard two-wire serial protocol. A dedicated module was built for this function using an Arduino microcontroller with an onboard micro-SD card (Figure 3B; Feather M0 Adalogger, Adafruit). In brief, after biologgers were explanted, extracted from their case, and cleaned, a custom 3D-printed clamp with an 8-pin pogo connector was used to break out the biologger programming pins. The biologger was then inserted into the data dump module and data was transferred from the biologger memory module to the micro-SD card. Although we also created a wireless data retrieval utility, having a dedicated system to offload data was simpler. Once the data is retrieved, the biologger is ready to be reused, sparring only the soldered electrode connector which is met with epoxy during encasing.

#### Closed-loop control

The biologger is capable of performing low-latency detection of neural rhythms when specific criteria are met. The closed-loop mode begins with a base station (LP-CC2652R1, Texas Instruments) connecting to the biologger, which is uniquely identified through a static address. After connecting, the biologger fills a fixed-size rolling buffer with incoming single-channel EEG data. After initially filling the buffer with 2 s of data, a detection algorithm is performed every 100 ms. We focused on the SW frequency band (0.5–4 Hz) characteristic of NREM sleep. The detection algorithm begins by decomposing a Fourier transform and finding the center frequency (F_*c*_) and phase of the ongoing neural rhythm. If F_*c*_ falls within the SW band, the ratio between the SW band power and power in the 6–12 Hz (empirically derived) range meets the user-set threshold, and the max amplitude of the bandpass filtered SW data (0.5–4 Hz Butterworth) meets a user-set threshold, the algorithm continues. If F_*c*_ is detected as being greater than 2 Hz, the algorithm is rerun on only the tail-end (1-s worth) of data to increase temporal sensitivity. The biologger packages relevant metadata including time, trial number, F_*c*_, and the phase at F_*c*_ for wireless transmission (16 bytes total) to the base station.

When the base station receives the detection packet, it calculates a delay to play a 50 ms audio tone centered on a specific phase of F_*c*_. Thus, phase precise closed-loop audio stimulation is achieved. The audio tone (calibrated to 50 dB) is generated by a standalone pink noise generator (NOISE2, Electric Druid) and amplification circuit with a manual volume knob. A bicolor LED was placed in the video frame of an overhanging camera to coordinate *post hoc* analysis of trials. Sham trials that excluded audio occurred with a 10% probability and were indicated by the LED as red versus green to easily differentiate trial conditions in video.

Once the biologger sends the stimulation packet, it continues to collect peri-detection biopotential data for another 2 s. After the entire trial buffer (4 s) is full, the biologger sends the entire data stream to the base station and saves it with the trial metadata to a micro-SD card. Trial metadata and biopotential data were combined with video data using custom scripts in MATLAB to generate peri-stimulus plots, as well as videos to ensure device function and animal behavioral state.

### Biological interface

#### Electrodes

Electrodes are typically made of a single piece of solid wire using a variety of different anticorrosive, biocompatible metals and alloys (Geddes and Roeder, 2003). Solid wire has the advantage of low resistivity but can easily shear when bent only a few times. A multi-strand cable is worse yet, as air gaps between wires can wick, collect, and hold moisture which degrades performance. Therefore, we developed a method to create single-conductor, helical (i.e., wound) electrodes not entirely unlike those used in human pacemaker applications (see also, Open Source Instruments online methods). We used 36 gauge (0.127 mm) 80 nichrome wire (Master Wire Supply) as the electrode and a piece of 0.38 mm steel music wire to wrap the electrodes. The nichrome and music wire were autoclaved before constructing the electrodes. First, the nichrome wire was attached to the top of the music wire with hot glue. Once secured, the nichrome wire was wrapped around the music wire to create the helix at a rate of roughly 10 turns per cm, and for a length of 30 cm. The nichrome wire was then clipped at both ends of the music wire and slid off. A sterile syringe (3 ml) was then filled with medical-grade silicone (A-100, Factor II). In preparation, translucent heat shrink tubing (Flexible 1.17 mm, Alpha Wire) was cold sterilized in a glutaraldehyde solution (Wavicide 01, Medical Chemical Corporation) for 12 h and then air-dried. The helical nichrome was then inserted into the heat shrink tubing and cinched at one end using a blunt needle to which the syringe was attached (using a Luer-style twist lock) and then local heat was applied to this junction to create a temporary seal. The syringe was then depressed to force the silicone into the tube cavity covering about 50% of the electrode. The heat shrink was then pinched at the syringe junction so it could not slide off and heated toward the open end of the heat shrink with moderate force to elongate the tubing as it was heated, ultimately enclosing the electrode and forcing the silicone into the remaining end of the heat shrink tubing. After the silicone encapsulated the wire, the electrode unit was pulled off of the blunt needle (and syringe) and the heat shrink tube was sheared at its furthest end where silicone had not entered. The internal silicone was left to set for >24 h and each electrode unit was eventually cut in half to create two, 15 cm pieces that were soldered to the biologger electrode port.

#### Encasing and encapsulation

Electrodes were interfaced with the biologger using a soldered connection. A small piece of custom cut, 1 mm foam was placed on the bottom of the 3D-printed case to eliminate hard surface-to-surface interfaces and reduce internal movement. Once the biologger was situated in the main part of the case, it was enclosed from its bottom side using a 3D-printed disc and medical device epoxy (EA M-31CL, Loctite) around the disc’s circumference which was held tight during the drying period using Kapton tape. The electrodes were fed through a 3D-printed conduit which interfaced to the case’s main body. The conduit was epoxied to the main case and then epoxy was injected into the conduit area around the electrode connector and base of the electrodes to create a structural interface to resist moisture from wicking into the biologger case. Once the epoxy was set (24 h), 6 g of a two-part silicone elastomer (RTV-4020, Factor II) were mixed and then degassed at 30 in Hg for 2 min in a vacuum chamber (1.5-gallon chamber, Ablaze Custom). The silicone was then poured into a 3 ml syringe which was centrifuged (custom made) to consolidate the silicone toward the ejection side. With the biologger suspended from the tail-end of its electrodes, silicone was applied through the syringe and an 18-gauge needle from 1 cm above the electrode interface all the way down onto the whole of the biologger. Excess silicone was allowed to drip into a small, disposable cup below. The biologger was then covered with a bell jar and allowed to sit for >24 h.

At all times, sterile best practices were followed during device preparation and handling. Once the biologger silicone was set, and 12 h prior to surgery, the biologger and electrodes were cold sterilized in a glutaraldehyde solution, after which it was placed into a large, empty sterile syringe (60 ml) for transport to the operating room.

### Surgical procedures

All procedures were approved by the University of Michigan Institutional Animal Care and Use Committee (IACUC) and Unit for Laboratory Animal Medicine (ULAM) under protocol PRO00009223. Red squirrels (*Tamiasciurus hudsonicus*) and fox squirrels (*Sciurus niger*) were trapped at Saginaw Forest in Ann Arbor, MI, under the Department of Natural Resources (DNR) Scientific Collectors Permit SC-1650. We used both species of squirrel to assess acclimation to captivity and tolerance of surgical procedures, such that we use squirrels in plural to describe the procedures. However, we use a single case study of a fox squirrel (∼430 g) in our results. We obtained rats (∼250 g) from our institutional reuse program.

Animals were anesthetized using 5% isoflurane (item 402017, MWI Animal Health) mixed with pure oxygen (Cryogenic Gases) in a custom plexiglass induction chamber. We found that squirrels required an immediate dose of the sedative dexmedetomidine (item 502019, MWI Animal Health) at 100 μg/kg to tolerate the surgery. Thus, after moving animals to the stereotactic frame, rats were maintained at slightly higher levels of isoflurane (∼2%) than squirrels (∼1.25%) because of the sparing effects of dexmedetomidine.

Animals were given a pre-surgical analgesic of carprofen at 5 mg/kg (University of Michigan ULAM Pharmacy) and antibiotic cefazolin at 50 mg/ml (item 501099, MWI Animal Health). Internal body temperature was constantly monitored using a handheld small animal thermometer and regulated using an adjustable infrared warming pad (RT-0520, Kent Scientific). Eye ointment was applied, then the animals were shaved using handheld clippers. Animals were then secured using rat-sized ear bars in the stereotactic frame (model 963, Kopf Instruments) and repositioned in the gas mask (item 751859, Harvard Apparatus). Three alternating applications of rubbing alcohol and betadine were applied to the surgical area and then the surgical area was draped using plastic (Press’n Seal, GLAD).

A one-inch medial-lateral incision was made behind the neck and then a small posterior subcutaneous pocket was exposed and irrigated with sterile saline (item 5102245, MWI Animal Health) and kept hydrated with gauze. A half-inch anterior-posterior midline incision was made over the skull and then retracted to identify the bregma and lambda skull sutures. Although a squirrel brain atlas was not available, squirrels have roughly the same anatomical proportions as rats (see Paxinos and Watson, 2007). Four, 1 mm screw holes were placed in the skull over the left and right frontal cortex (AP: 1.5 mm, ML: ±1.5 mm, relative to bregma) for signal leads and the left and right cerebellum (AP: −1.5 mm, ML: ±1.5 mm, relative to lambda) for reference leads.

A subcutaneous route was established from the skull to the neck incision for the electrodes. All open sites were irrigated at least three times with sterile saline. Electrodes were first routed to the skull area and then the biologger was placed into the subcutaneous pocket. The posterior reference electrodes were clipped, stripped to expose 2 mm of wire, secured in the skull holes using a stainless-steel screw, and covered with a two-part cold-curing dental cement (Teets Cold Cure, item 525000, A-M Systems). Next, the frontal leads were secured using a similar method, and a smooth head cap was formed with the dental cement.

Incisions were closed using a 5-0 nylon suture (item M-N518R19, AD Surgical). Animals were tapered from isoflurane and placed on a recovery heating pad. Rats recovered in a standard, acrylic home cage until ambulatory, whereas squirrels were administered a 1 mg/kg dose of atipamezole (item 032800, MWI Animal Health) to reverse the dexmedetomidine, and allowed to recover in their home cage. For at least 1 day following surgery, rats received an injection of carprofen at 5 mg/kg and a similar dose was mixed in nut butter and offered to squirrels.

#### Housing

Rats were housed in standard rodent cages, received *ad libitum* water and chow (5L0B, LabDiet), and were provided enrichment items. Squirrels were housed in a larger cage made for small primates that included naturalistic elements (e.g., sticks and grass) as well as a custom-made nest box (9 cu. in.). Squirrels were also given *ad libitum* water and chow, but they were fed a mixture of nuts, seeds, and fresh berries to replicate their natural diet. Cages were recorded using two video cameras (Wyze Cam v3). All animals were kept on a standard 12-h light-dark cycle (coordinated with ambient conditions) and for squirrels, the temperature was reduced to 60°F.

### Metrics and statistics

#### Biologger latencies

To determine algorithmic latency, we extracted the relevant code and placed two C-code statements (*Clock_getTicks*, accurate to 10 μs) around the algorithm block and ran it 1,000 times, storing each calculated latency to a 32-bit unsigned integer array. The latency array was then exported to a binary file and imported into MATLAB to generate the latency metrics.

To determine BLE wireless latency, we physically connected a biologger to a base station with a synchronization cable. The biologger sent a pulse following the statement that initiated a wireless indication (16-bytes) to the base station. The base station detected the pulse through a hardware interrupt that reset a clock variable (using *Clock_getTicks*). When the base station registered the wireless indication, the clock was re-referenced, and the time difference was calculated and analyzed in a similar manner to algorithm latency using 1,000 trials.

#### Sleep cycle

The ebb and flow of SW sleep characterized by 0.5–4 Hz oscillations—sometimes called NREM, or stage N3 sleep—can be used to determine a sleep cycle (Patel et al., 2022). To obtain a single SW power vector, we quantified SW power magnitude over time using a spectrogram (MATLAB, *pspectrum*) and averaged it across the frequency dimension. We tested for a cyclical component of these data using a standard power spectrum. To determine if the resulting power spectrum data was not due to chance alone, we randomly permuted the SW power magnitude time series (*n* = 10,000 surrogates), recalculated the power spectrum for each, and then tested how often each frequency bin for the actual power spectrum was larger than the surrogate distribution.

## Results

### Closed-loop auditory stimulation

We implanted our biologger in rats (*Rattus norvegicus*) for up to 7 days to assess the biological compatibility of small rodents and demonstrate utility in closed-loop experiments. We collected biopotential data at 125 Hz and performed real-time detection of SWs including information about the dominant (or, center) frequency and instantaneous phase (the “algorithm,” see section “Materials and methods” and Supplementary Figure 1). Although parameters will vary depending on the type of experiment, we suggest that algorithm runtime and wireless latency are relatively ubiquitous “fixed costs,” and important to characterize so that they can be included as line delays in the closed-loop control system. Algorithm runtime on our entire input signal (2 s of data) was 20.06 ± 0.01 ms (*n* = 1,000 trials). When SWs occurred at higher frequencies (>2 Hz), our algorithm reanalyzed only the tail portion of the data stream to increase detection specificity, resulting in an aggregate runtime of 25.55 ± 0.01 ms (*n* = 1,000 trials; an additional cost of 5.49 ms). Note that both devices—the peripheral (biologger) and central—handshake on a 10 ms connection interval prior to engaging in any further communication, but that the BLE protocol contains immutable random jitter to avoid packet collisions from multiple devices. We found our wireless latency to be 10.17 ± 1.74 ms (*n* = 1,000 trials), congruent with the intended connection interval. Only 0.3% of our wireless transmissions were outside three SDs of the mean with the largest difference being a single trial with a latency of 24.54 ms. These results may vary based on signal strength and 2.4 GHz band congestion.

**FIGURE 4:**
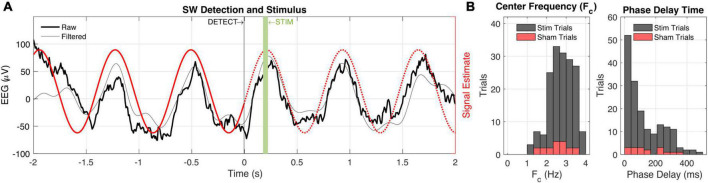
Closed-loop slow-wave activity detection and audio stimulation. **(A)** Peri-detection EEG data are shown (black) with a 0.5–4 Hz bandpass filter applied to better visualize slow-wave (SW) activity. The signal estimate (red) is based on the center frequency and phase estimation of the biologger which is relayed to the base station at *t* = 0. The base station subsequently estimates a phase delay time to play 50 ms audio stimulus at the up-going phase of the ongoing SW activity. **(B)** Session-wide (*n* = 202 trials) values for center frequency (Fc, left) and phase delay time (right). Sham trial distributions (10% probability) are shown in red.

Taken together, we were able to estimate the future signal of ongoing biopotentials with high accuracy by quantifying and anticipating system delays (Figure 4). Practically, from detection on the biologger to an audio stimulus from the wireless base station, we incur a 30° lag given a 2.5 Hz SW, and when that is accounted for, we obtain precision to within 1.5°. However, it should be appreciated that many biopotentials, and specifically neural EEG, rarely oscillate for many cycles at the same frequency, such that signal estimation is prone to error based on the location of recording, behavior being engaged, and organism itself.

### Approximating a free-ranging deployment

**FIGURE 5:**
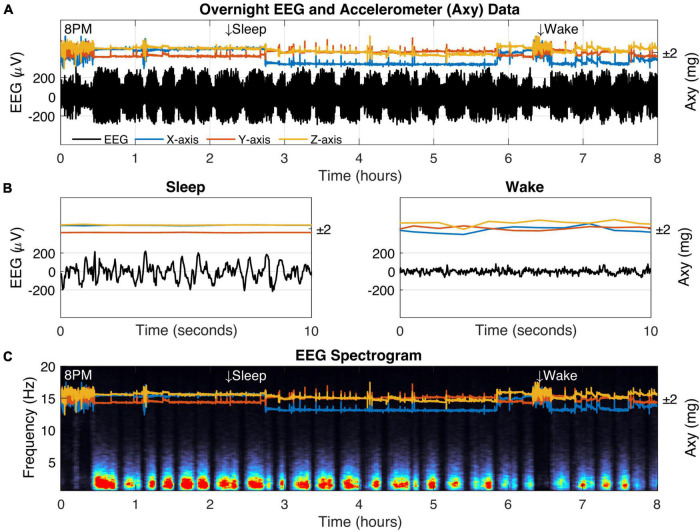
Biologging overnight in a freely behaving captive fox squirrel. **(A)** Eight hours of biologger data beginning at 8 p.m. showing EEG data (black) and 3D accelerometer data from the *x*-axis (blue), *y*-axis (orange), and *z*-axis (yellow). Representative sleep and wake epochs are marked along the top and the same data is shown in where **(B)** time has been restricted to a 10-s window. **(C)** The EEG spectrogram showing the relative power for each frequency (1–20 Hz) across time (red colors indicate high power).

We implanted a fox squirrel (*S. niger*) with our biologger to demonstrate the ability to obtain freely behaving behavior and physiology in a diurnal rodent (Figure 5). Our biologging recording utility includes the ability to modify the duty cycle of recordings, which are saved on-board. Here, we used a 100% duty cycle to record a full night’s worth of biopotential (sampled at 125 Hz) and accelerometer data (sampled at 1 Hz). We demonstrated the ability to retrieve these data for *post hoc* analysis through a biologger explant and humanely return the squirrel to its home territory in the forest.

**FIGURE 6:**
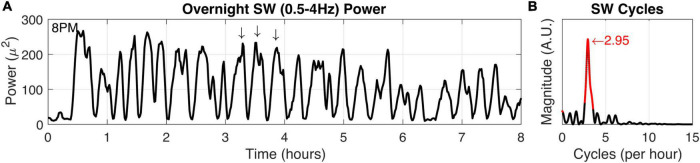
Squirrel SW cycle duration. **(A)** Power in the SW band (0.5–4 Hz) was calculated from overnight recording data (8 h). Arrows indicate high SW power and also demonstrate characteristic SW cycle frequency in a 1-h window. **(B)** The SW time-frequency relationship was calculated to determine fundamental frequencies that may occur in the SW activity. Highly significant (*P* < 0.001) values and the peak magnitude were calculated (both in red).

We verified squirrel behavior using an overhead nest camera (Supplementary Figure 2). It was evident that SW activity dominates the neural EEG when movement ceases, consistent with NREM sleep (Figures 5B,C). Overall, SW activity presented regularly throughout the night in our squirrel. We further demonstrated the utility of high-fidelity physiology by assessing the SW cycle length (i.e., sleep cycle) from these data (Figure 6). Our squirrel had a sleep cycle of 2.95 cycles per hour (*P* < 0.001), or roughly 20 min.

## Discussion

We developed and then deployed a wireless biologger in two experimental paradigms. Firstly, we showed that a closed-loop control system can be built around on-board algorithmic capabilities and low-latency wireless communication. Secondly, we demonstrated multi-day biologger durability in a freely behaving squirrel. Beyond technical or experimental capability, our biologgers were reused without incident, sparing only a few parts. We estimated that our devices had a one-time use cost of $120 (at quantity 25), a price point that enables large-scale deployments where neurophysiology is typically cost prohibitive. Taken together, our biologger device represents a single platform to support free-living neurophysiology that can be interacted with in real time in a laboratory setting or deployed autonomously in the field.

We propose that low-cost, implantable biologgers are one solution to approaching outstanding questions relating to the evolution of sleep. Squirrels are one such animal where rich data exist regarding social structure, waking behavior, and energetics (Boon et al., 2008; Fletcher et al., 2014; Studd et al., 2019, 2020; Dantzer et al., 2020), yet how squirrels sleep in the wild remains largely uncharted (but see Walker et al., 1977; Krilowicz et al., 1988). Although we (and others) have identified that squirrels sacrifice rest during autumn when storing food is critical to survival (Williams et al., 2016; Gaidica et al., 2021), it is unclear if sleep adapts to cope with, or enable such extreme fluctuations in waking behavior. We showed here that SW activity cycles roughly every 20 min in captivity which may be a useful parameter to evaluate in natural conditions given the strong relationship (found mostly in laboratory settings) between SWs, memory, and fitness (O’Hearn, 2021). Squirrels also highlight issues related to quiescent behavioral states indistinguishable from sleep as measured by movement alone (Halsey et al., 2009; Brown et al., 2013). We clearly observed extended bouts of active rest (e.g., motionless but eyes-open) in our captive squirrel. This is an effect known as “masking,” where animals like squirrels will use the confines of their nest not only for sleep but to mitigate exposure to weather and predators (Rietveld et al., 1993). Masking events are, therefore, challenging to discern through inertial sensors alone because they depend on stochastic environmental conditions often covert to the observer (Brown et al., 2013). Since many of the questions regarding the function of sleep depend on the prevalence and precise timing of sleep states, it is hard to imagine how those would be sufficiently addressed without neurophysiology.

The ability to implant a neural recording device opens several avenues to behavioral neuroscientists interested in fieldwork. For example, contact tracing has become a popular method by which individual interactions can be quantified (Berkvens et al., 2019) but there are few instances that describe changes in brain activity during socializing (but see Zhang and Yartsev, 2019) or conflicts in the wild. Unencumbered movement made possible by an implantable may also usher naturalistic problem-solving tasks that can probe intra- and inter-species cognition. Leaver et al. (2020) demonstrated an interaction between paw preference and learning in a novel paw preference test with wild gray squirrels which could be supplemented by understanding the neural correlates of paw preference and reaching dynamics (Bova et al., 2020).

The species candidate pool for neuro-biologging is going to be fundamentally limited by battery power into the future (Laske et al., 2021). While semiconductor size will continue to decrease exponentially (halving every 18 months), battery technology has failed to keep pace, ultimately playing into ethical concerns regarding the “5% rule”: a device should never exceed 5% of an animal’s mass (Portugal and White, 2018; Kay et al., 2019). Smaller batteries not only decrease in capacity, but our tests show that they are incapable of providing stable power (e.g., 2016 style battery), likely due to the high transient demands of a wireless radio and bio-amplifier. Although power efficiency is increasing in this device class, it will remain a challenge over the next several years to record continuously from neural biologgers for more than a few days in small animals (<50 grams). One potential solution that we explored with our biologger configuration utility is to subsample physiological data through duty-cycled (i.e., scheduled) recording routines, which can still provide a wealth of data tailored to a set experimental duration (van Hasselt et al., 2021). However, the growing use cases of the coin-cell form factor, including the ability to use rechargeable batteries (see Musk, 2019 and Neuralink), and the implementation of integrated regenerative sources (Iyer et al., 2022), is promising, but also depends on the experimental paradigm and access to the animal (Kim et al., 2021; Wright et al., 2022).

More research is needed to address the efficacy of releasing an animal into their natural habitat with a neural biologger, especially if it requires multiple procedures and a survival endpoint (Rattenborg et al., 2008). Some meta-analyses (mostly in birds and marine mammals) do show that biologgers can have slight but statistically significant negative impacts on animals (Barron et al., 2010; Bodey et al., 2018) whereas others do not (Bridge et al., 2013). Current evidence suggests that implantation of biologgers may be the better option compared to the external attachment as implanted devices do not seem to have long-lasting impacts on natural behavior, markers of inflammation, or detrimental effects on survival and reproduction in free-living animals (White et al., 2012; Shuert et al., 2015; Horning et al., 2017; Lameris and Kleyheeg, 2017; Forin-Wiart et al., 2019; Won et al., 2020). Meta-analyses have consistently shown that the method of attachment of a biologger influences the costs with external tags having the most detrimental effects and implanted devices having few significant impacts (White et al., 2012; Bodey et al., 2018). External attachment of biologgers may carry such costs because they increase the risk of entanglement, visual conspicuousness to predators, or increase costs of movement due to drag produced by the devices, whereas implantables promote freedom of movement and natural kinematics while maintaining the integrity of animal skin or fur allowing for natural interactions and grooming (Forin-Wiart et al., 2019). However, extended post-surgical observation that typically benefits an animal may lead to territories being overtaken or resources being poached in some species (Hendrix et al., 2020) and temporary removal of an animal may also have direct and indirect consequences on parental care or social dynamics in an ecosystem (Bulla et al., 2019). Detailed studies in the laboratory also show that any costs of implanted bio-loggers are short-lived as long as the total mass of the device does not exceed 3–5% of the total body mass of an individual (Casper, 2009). For example, a bio-telemetry device that was implanted in rats that were ∼1% of their average body mass had no impact on post-implantation body mass and the impacts on their behavior only lasted 2 days after surgery (Leon et al., 2004). However, Bakker et al. (2014) found that recovery of more complex motor behaviors following a neural implant in a non-human primate took 8–31 days.

There were clear advantages of performing this work on campus with veterinarian oversight. For example, we noticed mild seroma (i.e., fluid) development at the bio-logger implant site in in our early rat surgeries that was determined to be bacterial. To offer additional antibiotic coverage, we were able to quickly adjust our protocol—from using enrofloxacin to cefazolin—after which, we experienced no seromas and completely healthy surgical recovery in rats and squirrels (see also McCauley and Wehrens, 2010; Wedel et al., 2014). These refinements work toward the capability of performing bio-logger implants at a field station; the main considerations being the maintenance of a sterile surgical field, portable equipment and consumables, and suitable recovery and animal housing areas. Researchers should be prepared to work closely with their veterinarians and IACUC to establish suitable offsite procedures suited for the species of interest.

## Conclusion

In conclusion, the ability to record neural electrophysiology in a freely behaving animal, compute and communicate wirelessly with low-latency, and reduce deployment costs to enable large cohort studies are major hurdles we sought to address with our biologger. Despite some outstanding challenges, these capabilities have the potential to reveal novel insights while expanding the repertoire of species from which our scientific assumptions are built upon.

## Data availability statement

The datasets presented in this study can be found in online repositories. The names of the repository/repositories and accession number(s) can be found in the article/Supplementary material.

## Ethics statement

The animal study was reviewed and approved by the University of Michigan Institutional Animal Care and Use Committee (IACUC) and Unit for Laboratory Animal Medicine (ULAM).

## Author contributions

MG was the primary scientist and engineer. BD provided mentorship and expert feedback on approach and implementation. MG and BD wrote the manuscript. Both authors contributed to the article and approved the submitted version.
