# Supplementary material for: An implantable neurophysiology platform: Broadening research capabilities in free-living and non-traditional animals
Source: Front Neural Circuits. 2022 Sep 23;16:940989. doi: 10.3389/fncir.2022.940989 (PMC9537467; doi:10.3389/fncir.2022.940989)
Supplement: Supplementary file 1 [file Data_Sheet_1.docx]

Supplementary Material

# Supplementary Figures

| A | 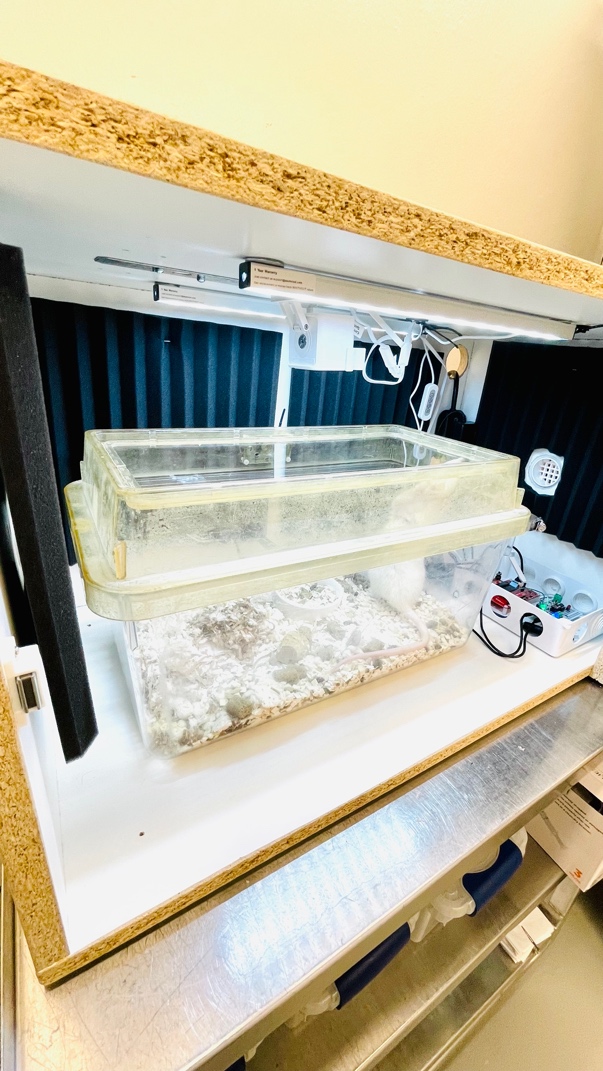 | B | 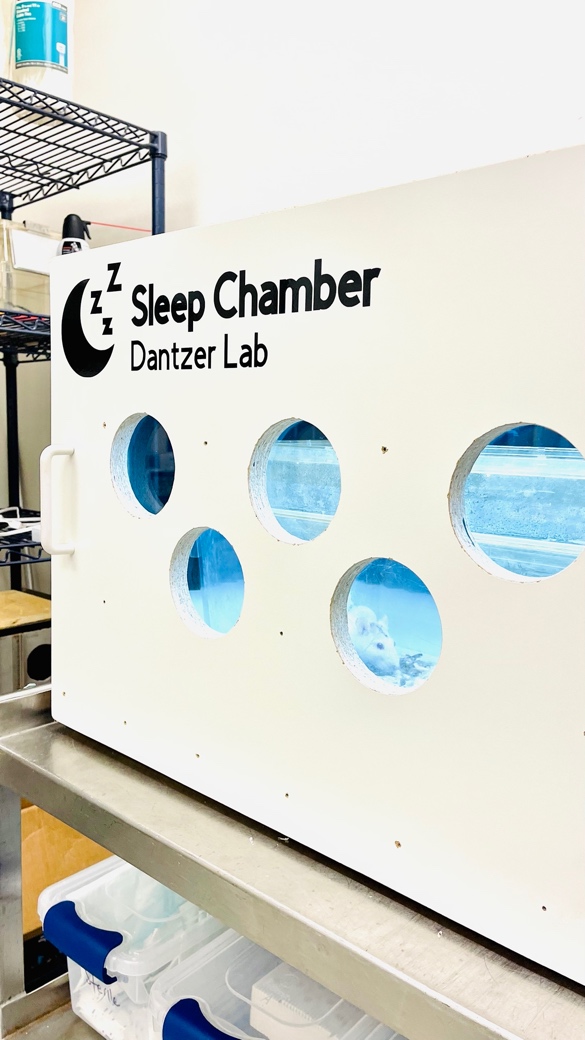 |
| --- | --- | --- | --- |

**Supplementary Figure 1.** Slow-wave (SW) Closed-loop Audio Stimulation Chamber. **(A)** The rat home cage was modified with a clear cage-top for the overhead video camera with a speaker attached to the lip (partially visible). Overhead lighting ensured proper light cycles were maintained in the chamber. The SW audio base station (lower-right) was in view of a second overhead video camera to monitor experimental progress. **(B)** The chamber closed from the front eliminating external noises and included transparent acrylic viewing holes as well as a low-noise fan and ventilation ports.

| A | 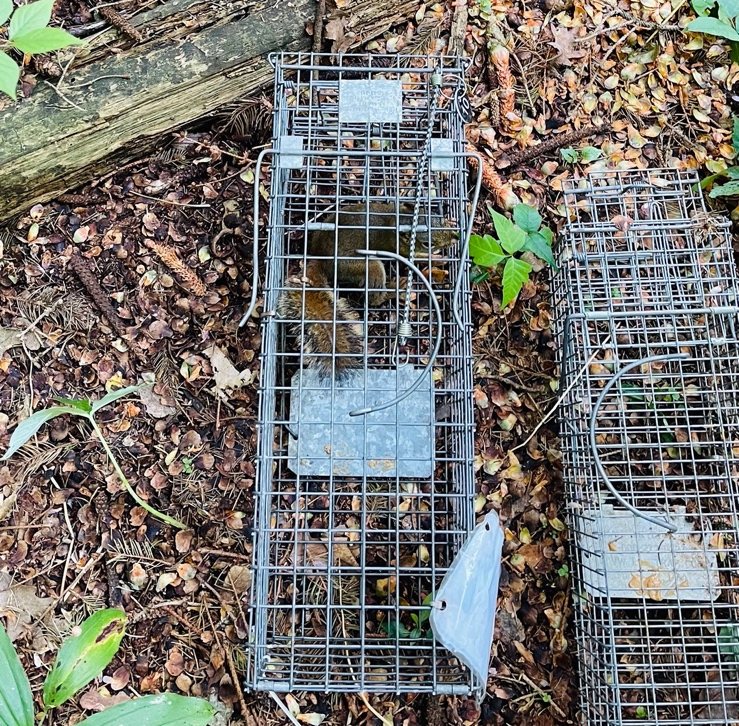 | B | 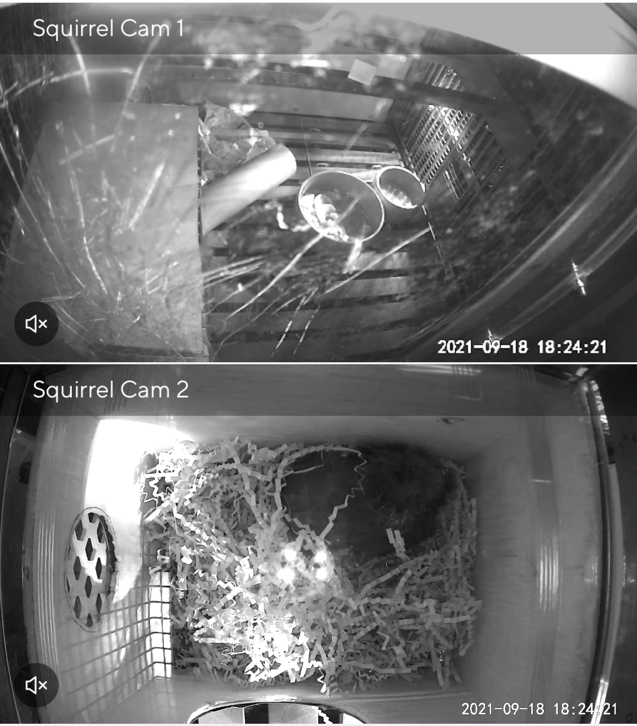 |
| --- | --- | --- | --- |

**Supplementary Figure 2.** Trapping and Monitoring a Squirrel. **(A)** A fox squirrel was trapped in a standard 1” wire cage trap using peanut butter bait. The squirrel was then transported to the University of Michigan campus for temporary housing. **(B)** A modified small primate cage was used to house the squirrel which included natural food and multiple water sources, as well as natural enrichment and bedding materials (e.g., sticks, hay/grass, cardboard tubing, cotton). A nest box was placed in the cage with an overhead camera and another camera was externally mounted that enabled real-time monitoring of the squirrel with timestamps to sync with the biologger recordings.

# Digital Repositories

All data, code, and designs are publicly available on GitHub: [https://github.com/mattgaidica/ESLO- Methods](https://github.com/mattgaidica/ESLO-Methods).
